# Puerperal Convulsions

**Published:** 1870-02

**Authors:** J. Little

**Affiliations:** Leroy, Illinois


ARTICLE III.—*Puerperal Convulsions:* An essay read before the McLean County Medical Society, October, 1869. By J. LITTLE, M.D., of Leroy, Illinois.

NOTHING is more terrifying to a family than to have one of its members seized with convulsions; and never is a physician more hastily and earnestly appealed to for prompt aid than on such occasions. And in no class of cases has he so little time to reflect; and hence the importance of being well informed, and knowing the very best course to be pursued in the treatment of the different kinds of convulsions.

The term convulsion may be defined, "a violent, irregular, and involuntary contraction of muscles ordinarily under the control of the will." The phenomena and pathology of convulsions are essentially the same in all cases, though the causes and symptoms may be dissimilar. Convulsions are said to be tonic and clonic, depending on the duration of the spasm; tetanus belongs to the former and epilepsy to the latter variety. Clonic convulsions are simple in character; epileptic, epileptoid, and puerperal convulsions belong to this division.

The causes of convulsions may act centrically or eccentrically; centrically when they act upon the nervous centres primarily, and eccentrically when they act on the nerves of some organ or part, and are then reflected to the spinal cord. When a woman has convulsions during, previous to, or after parturition, they are called puerperal. In one sense this is always true, but in another sense it is not, perhaps, always true. When we speak of puerperal convulsions, we mean convulsions depending *per se* on the puerperal condition, or convulsions peculiar to the pregnant woman, generally occurring about the time of delivery.

Puerperal convulsions are not common—occurring in every 600th to every 1000th case. They are far more common in the primipara than in the multipara; more common, as a general rule, in the more respectable unmarried primiparæ than in any other class.

Clinical observation has demonstrated that women whose urine is albuminous are very liable to have convulsions, and those whose urine is free from albumen are exempt. And it has further proven, that in proportion to the richness of albumen is the danger of convulsions increased. It has also proven, that if the albumen can be made to disappear from the urine, the danger of convulsions is passed.

But, is albumen in the urine a cause of puerperal convulsions? By no means. It only shows a disease of the kidneys. General or local anasarca, pallor of the skin, vertigo and feebleness, are symptoms of albumen in the urine; but, when suspected, the physician should be content with nothing less than its detection in the urine by nitric acid and heat. Now, when the kidneys are so diseased as to pour out with the urine the albumen of the blood, the urea is not eliminated from the system, but accumulates in the blood, and causes convulsions. Urea is an excrementitious principle, and in health is always found in the urine in small quantity; but when, from disease or any other cause, it is not excreted from the blood by the kidneys, it produces toxæmiæ of the most dangerous kind.

In Bright's disease there is albumen in the urine, and an excess of urea in the blood, and uræmic convulsions frequently occur. If one or both kidneys be removed from a dog, he will die of uræmic convulsions in two or three days. Now, what causes the condition of things of which we have just spoken, and which portends convulsions? Acute Bright's disease. And what causes this? Pressure of the gravid uterus upon the kidneys. Pressure of the gravid uterus causes frequently an enormous accumulation of fæces in the large intestine, and this increases the pressure on the kidneys. Pressure of the pregnant womb causes the veins of the lower extremities to be over-distended, and hence the cellular infiltration; and it prevents the usual amount of arterial blood going to the lower extremities, and causes an undue amount to be sent to the head and trunk and upper extremities, and causes a sur-excitation of the great nerve-centres, rendering the individual more susceptible to abnormal influences.

Taking all the facts into consideration — that primapara are more liable to puerperal convulsions than multipara; and that they are more liable because the pressure on the kidneys and gread blood vessels is greater, on account of tight lacing, to which they resort to conceal their condition, and their abdominal walls being more rigid than women who have borne children; the pressure of albumen in the urine, and urea in the blood, and the similarity of puerperal convulsions to pure uræmic convulsions — we must conclude that the great exciting element in puerperal convulsions is urea. Then the most successful treatment proves this conclusion to be correct.

When the kidneys are disabled, the skin and bowels are the channels that afford the most natural and direct outlet for the excrementitious elements of the urine. Saline purgatives and diaphoretics of the most active class, is the approved practice in warding off a threatened attack and in treating a case of puerperal convulsions. When the urea cannot be eliminated, on account of the frequency or severity of the convulsions, the next best treatment is to weaken its power, or render the parts upon which it acts less susceptible to its influence. Blood-letting, if the subject will bear it, will weaken the action of the poison by lessening its quantity in the blood, and chloroforming the patient will destroy the susceptibility of the nerve-centres to the urea. If all other means fail, the pressure on the kidneys and abdominal blood-vessels must be removed by prompt delivery, if possible.

Convulsions in the pregnant female, as in any other person, may be caused by acrid or indigestible substances in the stomach or bowels, and their cure demands the immediate removal of the cause. The pressure of the child's head, in its passage through the pelvic straits, may cause convulsions, but, as soon as the head is delivered, the convulsions cease. An over-distended bladder or bowel, a great and sudden impression, may cause convulsions in a parturient woman. These causes act eccentrically, and the convulsions so produced are not, as before intimated, really puerperal convulsions, because convulsions in children, or men, or the non-pregnant female, are produced by such causes.

The principal signs of puerperal convulsions are, albumen in the urine, urea in the blood, and local or general anasarca. The chief symptoms are, headache, vertigo, constipation, bad taste in the mouth, nausea, amaurosis, double vision, muscæ volitantes, listlessness, sharp or severe pain in stomach, back, bowels or head, jactitation, etc. A general uneasiness and restlessness often precede for some hours and usher in the convulsions.

In all cases where we have the least grounds for suspecting convulsions, it is our duty to examine the urine at once, and if albumen is present, to use prophylactic treatment until it disappears. The most successful preventive measures are saline cathartics, diaphoretics, diuretics, and a non-nitrogenous diet. The woman should be directed to take outdoor exercise on foot, to lie on her sides, and to leave off her corsets and all close-fitting garments, in order to relieve the kidneys from pressure. There is no reasonable doubt that almost all cases of puerperal convulsions might be prevented by a rational course of prophylactic treatment timely begun. In this, as in many other disorders, the preventive treatment is the most successful, and, therefore, the most important. But the child-bearing part of community is not aware of this, and it is the physician's duty, in his rounds, to look out for cases requiring prophylactic remedies and prescribe them.

But when puerperal convulsions have occurred, the most successful treatment seems to consist in a judicious use of chloroform, blood-letting, stimulating enemata, hot vapor baths, cold to head, dry and wet cupping over the region of the kidneys and on the nape of the neck, or strong mustard drafts, and active saline cathartics or croton oil. Colchicum, benzoic acid, and acetate of potassa are the most highly recommended as diuretics; but these cannot be relied on to the exclusion of other eliminatives, as the kidneys are generally too much crippled to respond to diuretics. Bromide of potassium is highly recommended by many who have used it with good success. It is thought to act on and through the nerve centres, lessening cerebral and spinal congestion, and producing an anodyne and soporific effect, thus destroying, to some extent, the susceptibility of the nervous system to the spasm-producing agent. When every thing else fails, if circumstances would at all justify, the womb should be emptied, as the child in the uterus, and the urea in the blood, are the causes of puerperal convulsions. But if the urea can be eliminated or neutralized, or if the parts on which it acts can be so affected as to be insensible to its action, by treatment, the convulsions will be cured.

If our views as regards the causation and pathology of puerperal convulsions are correct, their treatment, to be rational, must eliminate or neutralize the urea, or render the nerve centres insensible to its presence. This, then, is the great object in the treatment of cases, whether occurring before, during, or after delivery. I have treated several cases of genuine puerperal convulsions, in accordance with the views here presented, with the best success, and will give the history and treatment of but a single case, in this connection, as it will serve to illustrate the foregoing remarks.

About eleven o'clock on the night of September 18, was called to visit Mrs. L., in haste, as the messenger said she was having fits. Was soon at her bedside, and obtained the following facts: She was taken in labor, at full term, on the evening of September 17, and after a tedious and harrassing labor was delivered of a large, healthy child, at ten and a half o'clock, A.M., on the 18th. She was a primipara, twenty-two years of age, nervo-sanguine temperament, stout, short and muscular, of sedentary habits, and habitually costive. Her feet and legs had been considerably swollen for several days, and she had complained of dizziness, and headache, and trouble in voiding her urine.

The labor being completed, she was bandaged and dressed, and every thing done that could be to promote her comfort; but still she complained of a good deal of pain in the region of the womb, and as time wore on, the pain increased and extended to her back and stomach. She became restless, and turned and tossed from side to side, in spite of admonitions to lie still, or she would hurt herself or babe, until the latter had to be removed for safety. About five o'clock the pain in her head became intense, and about six o'clock a very severe pain occurred in the right hypochondrium, and these pains continued with no abatement till about seven o'clock, when the first convulsion took place. The convulsions returned about every hour and a quarter, and she had her fifth one ten minutes after my arrival.

Ordered a sinapism over region of kidneys, another on nape of neck, and another on epigastrium; a hot, stimulating pediluvia, twelve hot bricks, enveloped in wet cloths, to be placed in the bed pretty close to the patient, and cold water to head. Gave fifteen grains bromide of potassium, and a teaspoonful of spts.

eth. nit., with five drops fld. ext. colchium, and in fifteen minutes gave a tablespoonful sulph. mag., in half tumbler cold water, and in fifteen minutes more administered an enema, consisting of two-thirds of a pint of warm soap-suds, a tablespoonful each of castor oil, spts. turpentine, and common salt. Introduced catheter, and drew off about four ounces of dark, offensive urine, loaded with albumen. Repeated the bromide of potassium, and sat down by my patient, with chloroform at hand, determined to forestall the next convulsion by its use; but in this I was disappointed, for, before I was aware of it, the time for its return had come, and she was seized with another hard convulsion. When it had passed away we persevered in the treatment begun, repeating all the internal remedies in the order mentioned above. About fifty minutes after the last convulsion her bowels moved copiously, her urine passed freely, and she was perspiring profusely. Ten minutes before the time for next convulsion she was chloroformed pretty thoroughly, and was kept in this condition, with an occasional letting up, for three hours. During this time her bowels and bladder had been well emptied again, and she had continued to sweat freely. I remained with her till eleven o'clock, A.M., on the 19th, continuing treatment less heroically than at first, and as she had had no convulsion since about two o'clock in the morning, and seemed calm, rational and comfortable, and could take a little nourishment, I pronounced her out of danger, and took my leave. Directed a half Seidlitz powder to be given twice a day, and a teaspoonful of this: ℞. — Spts. mindereri, f ℥ iiss; tinct. colchici. sem., f ℥ ss. M. To be given every four hours, for two or three days. Light, bland nourishment, three or four times a day, and cold lemonade *ad libitum*. Perspiration to be encouraged by warm sponge baths, and the warm bricks. Heard nothing from my patient till October 5, when I learned she was very well, and had been doing her work for five days.

